# Genomic Survey of *PEBP* Gene Family in Rice: Identification, Phylogenetic Analysis, and Expression Profiles in Organs and under Abiotic Stresses

**DOI:** 10.3390/plants11121576

**Published:** 2022-06-15

**Authors:** Chunyu Zhao, Mo Zhu, Yanying Guo, Jian Sun, Wenhong Ma, Xiaoxue Wang

**Affiliations:** 1 Rice Research Institute, Shenyang Agricultural University, Shenyang 110866, China; 2019220264@stu.syau.edu.cn (C.Z.); guoyy@stu.syau.edu.cn (Y.G.); sunjian811119@syau.edu.cn (J.S.); 1988500023@syau.edu.cn (W.M.); 2 Institute of Agricultural Biotechnology, Jilin Academy of Agricultural Sciences, Changchun 130033, China; zhumo8989@163.com

**Keywords:** OsPEBPs, OsFTLs, PEBP-domain-containing proteins, phylogenetic analysis, rice

## Abstract

Phosphatidylethanolamine-binding-protein (PEBP) domain-containing proteins play important roles in multiple developmental processes of plants; however, functions of few members in the *PEBP* gene family have been elucidated in rice and other crops. In this study, we found that twenty *OsPEBPs* genes identified in rice are not evenly distributed on the chromosomes. Four colinear pairs are identified, suggesting the duplication of *OsPEBPs* during evolution. The OsPEBPs are classified into six subgroups by phylogenetic analysis. The structure of all the *OsPEBP* genes and encoded proteins are similar. The 262 PEBP domain-containing proteins from crops are divided into six groups. The number of colinear pairs varies between rice and other crops. More than thirty *cis*-acting elements in the promoter region of *OsPEBPs* are discovered. Expression profiles of *OsPEBP* genes are differential. Most of the *OsPEBPs* expression can be regulated by NaCl, ABA, JA, and light, indicating that *OsPEBPs* may be involved in the control of the response to the environmental signals. These results lay sound foundation to further explore their functions in development of rice and crops.

## 1. Introduction

Phosphatidylethanolamine binding proteins (PEBPs) are phylogenetically conserved from prokaryote to eukaryote. In mammals, PEBPs play important roles in multiple cellular and biological processes, including cell growth and differentiation, proliferation, cell cycle, genomic stability, apoptosis, autophagy, mechanical and oxidative stress, and immunity [1,2,3]. Downregulation of PEBP1 leads to major diseases, such as cancer and Alzheimer’s disease. The crystal structure reveals that PEBP1 has a small accessible cavity which is the binding site for phosphorylethanolamine, phosphate, acetate, or cacodilate molecules [4,5]. PEBP1 physically interacts with kinases and GTPases to inhibiting their activity. PEBP1 inhibits the kinase activity of Raf1 in Raf-MEK-ERK to suppress metastasis [6,7]. PEBP1 regulates activities of several other protein kinases in NF-kB and PI3K/Akt/mTOR pathways to implicate in cancer [8,9,10]. In addition, PEBP1 is associated with several factors of Rho-GTPases to impact the formation of actin structures in cell adhesion and motility governing cell migration [11].

In plants, PEBP family is involved in flowering time control, seed dormancy, plant architecture determination, tuber formation, and sink–source allocation [12,13,14,15,16,17]. PEBP proteins act as key factors to regulate vegetative to reproductive phase transition or floral transition in plants [18,19,20,21]. In Arabidopsis (*Arabidopsis thaliana*), Flowering Locus T (FT) protein, a member of PEBP family, has been demonstrated to be the florigen to induce flowering [16,22,23,24,25]. While the other three PEBP-domain-containing proteins, including Terminal Flower 1 (TFL1), Arabidopsis Thaliana Centroradialis (ATC), and Brother of FT and TFL1 (BFT), repress flowering in Arabidopsis [20,25,26,27,28,29,30]. FT and TFL1 act as transcriptional co-activator and co-repressor, respectively [31,32]. In shoot apical meristem (SAM), FT and TFL1 are associated with Flowering Locus D (FD), a basic leucine zipper (bZIP) transcription factor, mediated by 14-3-3 proteins forming florigen activation complex (FAC) or florigen repression complex (FRC) to regulate flowering [22,23,33,34]. The antagonism between TFL1 and FT relies on competition for accession to FD at the target loci [31,35]. TFL1 and FT are distinguished by only a few amino acid substitutions [25,26,32,36]. A single amino acid change or mutation can convert FT into TFL1 and vice versa [32,36,37,38].

PEBPs are involved in floral transition in potato (*Solanum tuberosum*). StSP3D, an orthologue of FT, controls flowering time in potato [12]. In addition, PEBPs are also involved in tuber formation of potato. Tuberization is induced by StSP6A termed tuberigen, an ortholog of FT in potato [12,39,40]. In the stolon tip, StSP6A is associated with St14-3-3s and StFD-Like 1 (StFDL1) forming the tuberigen activation complex (TAC) to promote tuber formation [12,41]. Conversely, StSP5G and TFL1/CENTRORADIALIS (StCEN), two members of *PEBP* gene family in potato, act as repressors of tuber formation by acting against StSP6A in stolons [12,42].

PEBPs are also involved in regulation of sink–source relationships. Different classes of sugar transporters, such as Sugar Will Eventually be Exported Transporters (SWEETs) and Sucrose Transporters (SUTs), have been known for their roles in determining sink–source dynamics [43,44]. In potato, tubers are sink organs to store photoassimilation products. Leaves are the source organs for photosynthesis. In addition to tuberization, StSP6A interacts with and blocks the activity of SWEET11 to stimulate tuber swelling [17]. StCEN, a PEBP protein in potato, promotes tuber dormancy or suppresses tuber sprout growth. The outgrowth of tuber bud is correlated with the expression decrease in *StCEN* [45].

Rice (*Oryza sativa*) is one of the most important cereal food crops, feeding about half of the world population [46,47]. It is also a monocotyledon model system, which is a typical short-day (SD) plant [48,49]. Functions of several OsPEBPs in rice, such as Heading date 3a (Hd3a), Rice Flowering locus T 1 (RFT1), Rice CENTRORADIALIS 1-4 (OsRCN1-4), OsFTL10, and rice Mother of FT and TFL1 (OsMFT1), have been reported [24,33,34,50,51,52,53]. *Hd3a* and *RFT1* are the homolog of *FT* encoding florigen in rice [54]. Hd3a and RFT1 are synthesized in leaves and transported to the SAM through the phloem [24,52,55]. Moreover, Hd3a interacts with 14-3-3 protein and then forms a FAC with OsFD1 in the SAM to promote flowering [33]. The FAC promotes flowering by activating the expression of *OsMADS14* and *OsMADS15*, which encode APETALA1(AP1)/FRUITFULL (FUL)-like MADS domain proteins [33,52,56,57]. Similarly, the FRC is formed with a homolog of the TFL1 in rice, revealing direct competition between the two PEBPs for complex formation [34,35]. In addition, it has been reported that Hd3a protein accumulates in axillary meristems to promote branching requiring the formation of FAC, which is the process independent from strigolactone and FC1, a transcription factor that inhibits branching in rice [58]. Similar mechanisms are also found in other plant species, including tomato (*Solanum lycopersicum*), wheat (Triticum aestivum), barley (*Hordeum vulgare*), and maize (*Zea mays*), suggesting that this molecular module is widely conserved among higher plant species [41,59,60,61].

Taking together, PEBP-domain-containing proteins in plants play important roles in crop development. However, the functions and evolution of PEBP protein family in rice and other crops remain unclear. Here, we isolate twenty genes encoding PEBP-domain-containing proteins in rice, termed *OsPEBPs*. The position on chromosome, gene structures, domains, conserved motifs, and phylogenetic features of *OsPEBPs* genes in rice are studied. The expression of *OsPEBPs* genes in organs, developmental stages, and stress conditions are analyzed. Phylogenetic relationships between *OsPEBPs* and homologs in wild rice species, Arabidopsis, and other crops are investigated.

## 2. Results

### 2.1. Characterization of the PEBP Genes in Rice

To identify the *PEBP* genes in rice, full-length amino acid sequences of FT protein from Arabidopsis, Hd3a and RFT1 proteins from rice are applied to find homologs of OsPEBPs by using BLAST and Hidden Markov Model (HMM). Twenty OsPEBPs are identified in rice genome, which are designated according to the Rice Annotation Project Database (RAP-DB) information. Properties of *OsPEBP* gene family and the proteins encoded are summarized (Table S1).

**Figure 1:**
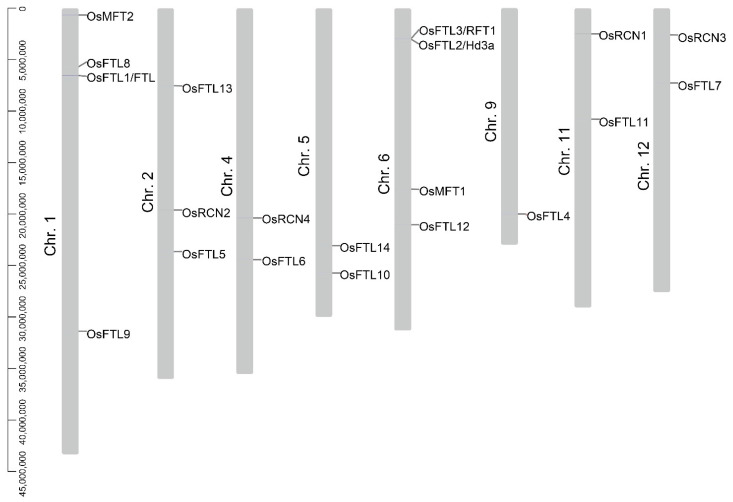
Distribution of *OsPEBP* genes on rice chromosomes. Bars in gray represent the rice chromosomes. The number on the left of the gray bars are the chromosome number. *OsPEBP* genes are labeled on the right of each chromosome. The ruler on the left is the scale bar measuring the length of chromosomes.

All of the *OsPEBP* genes were mapped to the rice chromosomes by using TBtools. The results show that the 20 *OsPEBPs* are not evenly distributed on the chromosomes (Figure 1). The *OsPEBPs* are positioned on eight chromosomes of rice. None of *OsPEBPs* are found in chromosome 3, 7, 8, and 10. The analysis reveals that some of the *OsPEBP* genes duplicate during evolution. *OsFTL8* and *OsFTL1/OsFTL* are close to each other on chromosome 1; *OsFTL2/Hd3a* and *OsFTL3/RFT1* are close to each other on chromosome 6, indicating tandem duplication of *OsPEBPs* occurs to form two small clusters during evolution (Figure 1).

Moreover, the chromosome length is not obviously correlated with the distribution of *OsPEBPs*. Although four *OsPEBPs* are positioned on the longest chromosome 1, four *OsPEBPs* are scatted on chromosome 6 which is shorter than chromosome 1 (Figure 1).

**Figure 2:**
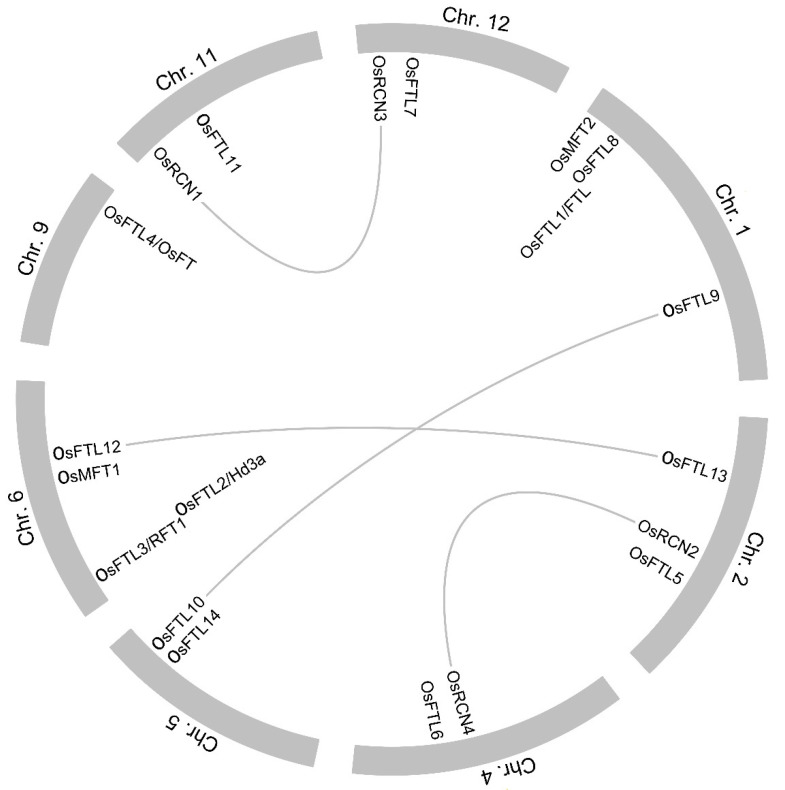
Syntenic analysis of *OsPEBP* genes in rice. Bars in gray show the rice chromosomes. Chromosome number is labeled on the peripheral surface. *OsPEBP* genes are labeled on the inner circumferential surface of each chromosome. The gray lines represent the colinear pairs of *OsPEBP* genes.

To clarify the evolutionary relationship of *OsPEBP* gene family, the syntenic relationship was analyzed by using whole-genome homology and gene positional information. The results show that four collinear pairs are found between *OsPEBPs* in rice, suggesting *OsPEBP* genes undergo segmental duplications (Figure 2).

### 2.2. Phylogenetic Analysis of the PEBP Genes in Rice

**Figure 3:**
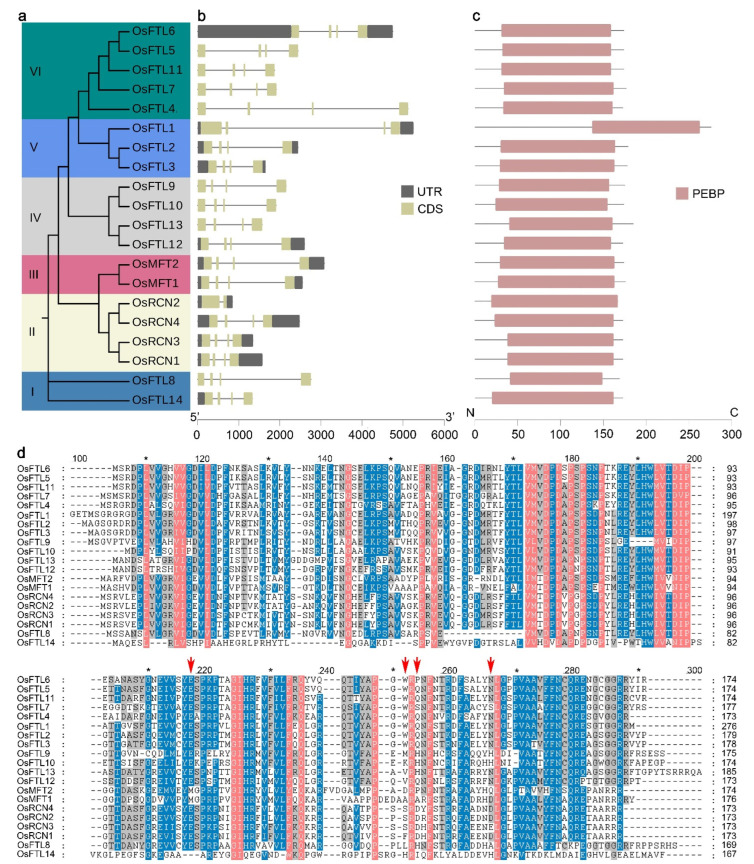
Phylogenetic tree and gene structure of *OsPEBP* in rice. (**a**) The phylogenetic tree of OsPEBPs. Different colors represent the subgroup of OsPEBPs. (**b**) *OsPEBP* gene structure. Black and yellow rectangles, and black lines indicate UTR, exon, and introns, respectively. UTR represents untranslated region. (**c**) Domains in OsPEBP protein. Pink rectangles represent PEBP domains. The rulers on the bottom of (**b**,**c**) are the scale bar measuring the length of PEBP genes and proteins. (**d**) Alignment of OsPEBP proteins. The red arrows indicate the potential key amino acid residuals of OsPEBPs.

To explore the evolution of OsPEBPs, we constructed the phylogenetic tree of OsPEBPs with their amino acid sequences. The results show that the members of OsPEBP family in rice are divided into six clades (Figure 3a). Clade I consists of OsFTL14 and OsFTL8. Clade II consists of OsRCN1 to OsRCN4. Clade III consists of OsMFT1 and OsMFT2. Clade IV consists of OsFTL9, OsFTL10, OsFTL12, and OsFTL13. Clade V consists of OsFTL1/FTL1, OsFTL2/Hd3a, and OsFTL3/RFT1. Clade VI consists of OsFTL4 to OsFTL7, and OsFTL11 (Figure 3a).

Intron gain and loss are common phenomena in evolution, which can increase the complexity of gene organization [62,63]. To examine the organization diversity of the *OsPEBP* genes, the genomic DNA sequence was compared against coding sequence to study the gene structure. All of the *OsPEBP* genes have three introns and four exons, except for *OsRCN2*, indicating that the gene structure of *OsPEBP* gene family is evolutionarily conserved (Figure 3b).

PEBPs in plants have diverse functions. An amino acid substitution in their C-terminals is able to change their functions [32,36,37,38]. To elucidate the relationship of amino acid sequence and their functions, the feature of OsPEBP was analyzed. All of the OsPEBPs contain a PEBP domain (Figure 3c). The alignment of OsPEBP protein sequences shows that the PEBP domain is conserved among OsPEBPs in rice. The key residuals in PEBPs’ C-terminal, including Glu217 (E217), Trp252 (W252), Gln254 (Q254), and Asn266 (N266), varies in the amino acid sequences of OsPEBPs, implying functional differentiation (Figure 3d).

### 2.3. Phylogenetic Analysis and Conserved Motifs of PEBP Proteins in Crops

**Figure 4:**
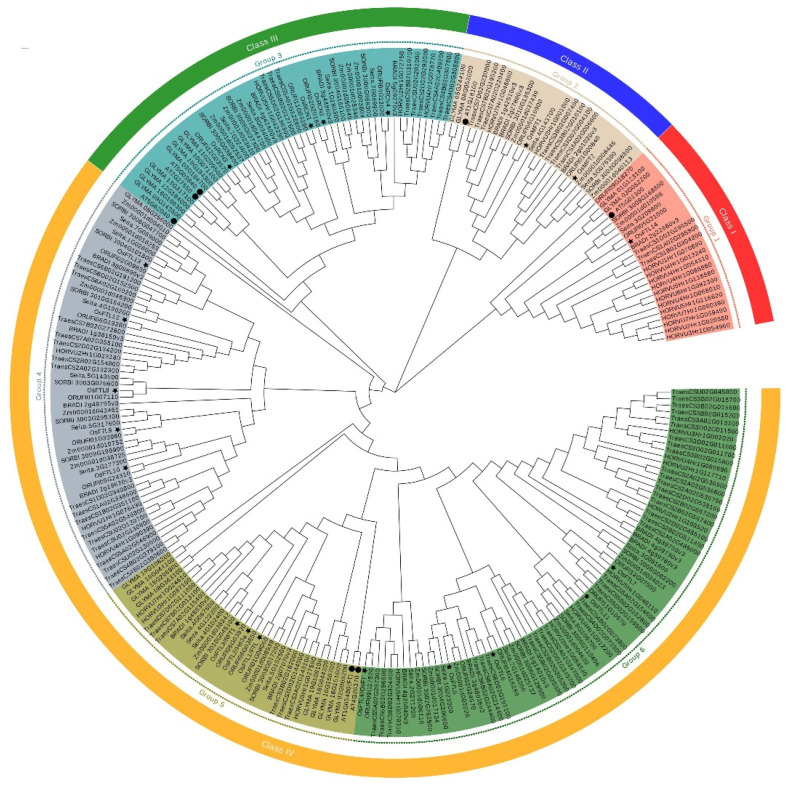
Phylogenetic analysis of PEBP-domain-containing proteins from crops. Phylogenetic tree among 262 PEBP proteins from rice, maize, wheat, sorghum (*Sorghum bicolor*), barley, millet (*Setaria italica*), brachypodium (*Brachypodium distachyon*), soybean (*Glycine* max), and Arabidopsis were constructed. PEBP proteins from rice and Arabidopsis are labeled by black stars and circles, respectively. The tree is constructed on the amino acid sequences of the PEBP proteins by MEGA-X with the maximum likelihood method. Bootstrap = 1000.

To reveal the phylogenetic relationships of PEBPs between rice and other crops, we constructed an unrooted phylogenetic tree using amino acid sequences of 262 PEBP proteins. The PEBPs are divided into six groups termed group 1 to 6, which belong to four classes, termed class I to IV (Figure 4). Group 1 to 3 are corresponding to class I to III, respectively. Group 4 to 6 are in class IV. OsFTL14 is in group 1. OsMFT1 and OsMFT2 are in group 2. OsRCN1 to OsRCN4 are in group 3. OsFTL8, OsFTL9, OsFTL10, OsFTL12, and OsFTL13 are in group 4. OsFTL1 to OsFTL3 are in group 5. OsFTL4 to OsFTL7, OsFTL11 are in group 6 (Figure 4).

**Figure 5:**
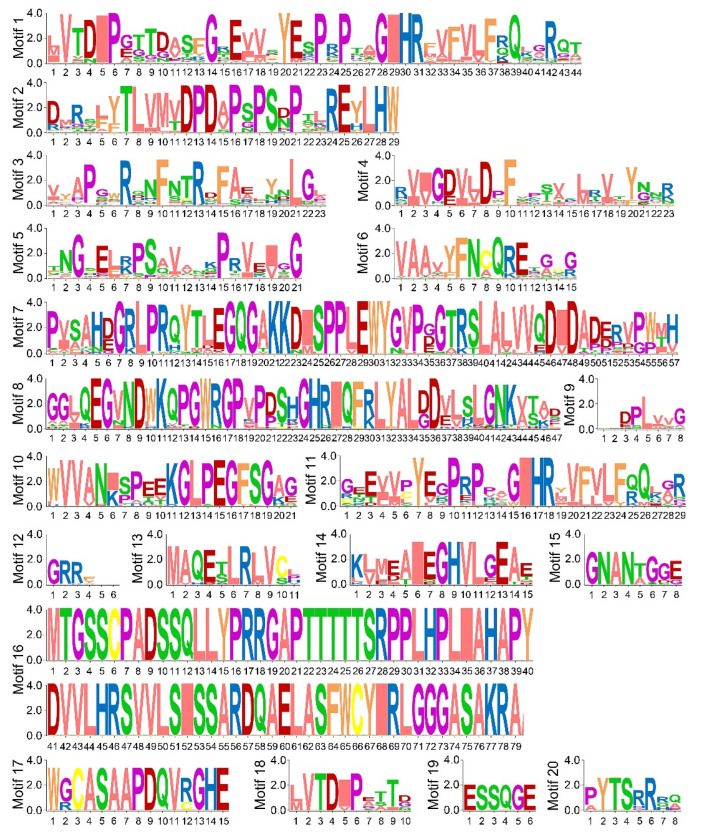
Motifs found in PEBP domain-containing proteins of crops. The height of different amino acids represents frequency of occurrence. The scale bar at the bottom indicates the length of the motif protein sequence.

To further study the features of PEBP proteins, conserved motifs in 262 PEBP proteins were analyzed by using Multiple Em for Motif Elicitation (MEME) in motif-based sequence analysis tools database. Twenty conserved motifs are identified among OsPEBP and PEBP proteins in other species. The sequences of the motifs identified are characterized (Figure 5). Motif 1 to motif 6, and motif 9 are present in the majority of PEBP proteins in group 2 to group 6. Motif 12 is in the most of PEBP proteins in group 4 and group 6. Motif 19 and motif 20 is in part of group 4 PEBP proteins (Figure S1). Motif 7, motif 8, motif 10, motif 13 to motif 15, motif 17 present in most of PEBP proteins in group 1. Only one copy of the motifs is identified in every single PEBP protein. Members in the same group possess similar number, orientation and position of motifs, indicating that the PEBP members in the same group may have similar functions (Figure S1).

### 2.4. Synteny Analysis of OsPEBP Genes between Rice and Crops

Synteny analysis is an effective way to explore the functional and evolutionary relationship between genes [62]. To further investigate the evolutionary relationship of PEBP proteins between rice and other crops, the syntenic relationship of PEBPs was analyzed.

**Figure 6:**
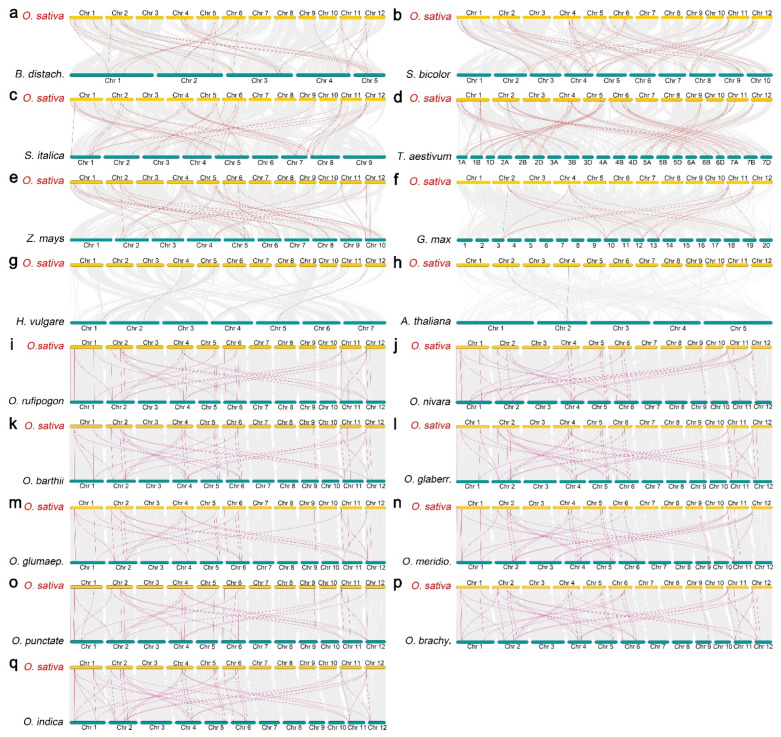
Syntenic analysis of *PEBP* genes between rice and other crops. (**a**–**h**) Collinear pairs between rice and crops, including brachypodium (**a**), sorgum (**b**), millet (**c**), wheat (**d**), maize (**e**), soybean (**f**), barley (**g**), and Arabidopsis (**h**). (**i**–**q**) Colinear pairs between rice and 8 wild rice species, including *Oryza rufipogon* (**i**), *Oryza nivara* (**j**), *Oryza barthii* (**k**)*, Oryza glaberrima* (**l**)*, Oryza glumaepatula* (**m**)*, Oryza meridionalis* (**n**)*, Oryza punctate* (**o**)*, Oryza brachyantha* (**p**)*,* and cultivated indica rice (*Oryza indica*) (**q**). The gray lines at the bottom represent the collinear regions in rice and other genomes. The red lines represent the pairs of *PEBP* genes.

Most loci near *PEBP* genes in rice present conserved synteny in most identified cereal species, including brachypodium, sorghum, millet, wheat, and maize. The number of colinear pairs between rice and the above crops is 31, 37, 37, 47, and 32, respectively (Figure 6a–e). These results suggest that the phylogenetic relationship between *PEBP* gene loci of rice and in brachypodium, sorghum, millet, wheat, and maize, is close. However, the collinear pairs of *PEBPs* between rice and the other species, such as soybean, barley, and Arabidopsis, is 19, 4, and 2, indicating the longer distance phylogenetic relationship between these species (Figure 6f–h).

In addition, the syntenic association was also analyzed between rice and rice ancestors, such as *Oryza rufipogon*, *Oryza nivara*, *Oryza barthii*, *Oryza glaberrima*, *Oryza glumaepatula*, *Oryza meridionalis*, *Oryza punctate*, *Oryza brachyantha*, and *Oryza indica.* The number of the collinear pairs of PEBP genes between rice and its ancestors is 38, 33, 38, 40, 34, 34, 35, 40, and 38, respectively, suggesting conservation of PEBP gene family and the significant roles during evolution (Figure 6i–q).

### 2.5. Cis-Acting Element and Gene Expression Analysis of the PEBP Genes of Rice under Stress Treatments

**Figure 7:**
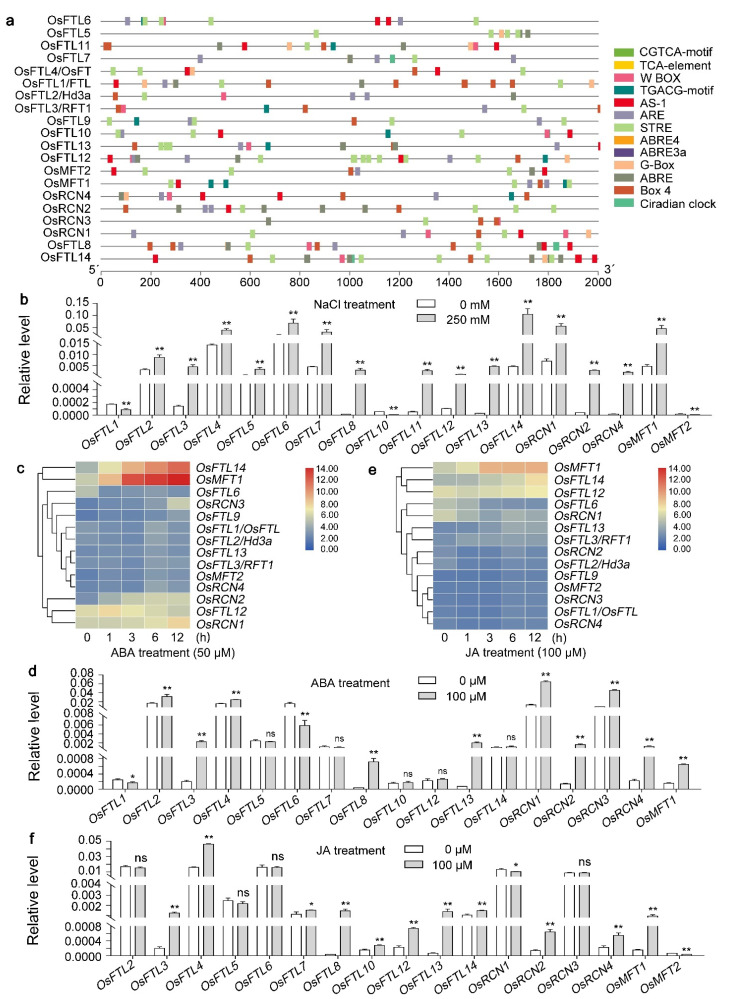
The *cis*-acting regulatory elements in the promoter regions of *OsPEBPs* and their functions. (**a**) *Cis*-acting elements identified in the *OsPEBP* promoter regions. Shapes and colors represent different *cis*-acting elements. Ruler on the bottom is used to the length of promoters. (**b**) Expression of *OsPEBPs* under NaCl treatment. (**c**,**d**) Expression of *OsPEBPs* under ABA treatment by microarray data (**c**) and qRT-PCR (**d**). (**e**,**f**) Expression of *OsPEBPs* under JA treatment by microarray data (**e**) and qRT-PCR (**f**). The blue to red color of the bars in (**c**,**d**) represents the lower to higher expression of *OsPEBPs*. Data in (**b**,**d**,**f**) are the mean ± SD. Statistic differences are analyzed by using two-tailed unpaired *t* test with Welch’s correction (*, *p* < 0.05; **, *p* < 0.01).

*Cis*-acting elements in promoters of genes are important to regulate gene expression. To better elucidate the mechanisms by which regulates the expression of *OsPEBPs*, the 2000 bp sequences upstream of *OsPEBPs*’ start codon were used as their promoters. The *cis*-acting regulatory elements in the promoter regions of *OsPEBPs* were analyzed by using PlantCARE database (http://bioinformatics.psb.ugent.be/webtools/plantcare/html/, accessed on 11 May 2021). More than 30 *cis*-acting elements are identified in the promoter regions of *OsPEBPs*, including 7 stress response elements, 6 light response elements, 14 hormone response elements, one anaerobic induction element, one seed-specific regulatory element, an O2-motif in zein metabolism regulation, and a circadian control element had been identified. Thirteen of them are shared by most of the *OsPEBPs*. The 13 shared *cis*-elements are mapped to the promoter region and distinguished by different colors (Figure 7a).

Stress response element (STRE) is the *cis*-acting element response to stresses [64]. The anaerobic regulatory element (ARE) is related with the anaerobic stress [65]. The STRE cis-acting element is identified in the promoters of all the *OsPEBP* genes, except for *OsRCN4*, indicating that OsPEBPs may be involved in the abiotic stress tolerance (Figure 7a). The ARE *cis*-acting element presents in most of the *OsPEBP* gene promoters, indicating that OsPEBPs may be involved in regulating the tolerance to anaerobic stress (Figure 7a). The expression of *OsPEBPs* under NaCl treatment were detected by qRT-PCR. The results show that most of the *OsPEBPs*’ expression is induced by NaCl, except for *OsFTL1*, *OsFTL10*, and *OsMFT2* (Figure 7b).

The abscisic acid (ABA) responsive element (ABRE), ABRE3a, and ABRE4 are the *cis*-acting elements response to ABA. As-1, W box, and TCA-motif are the *cis*-acting regulatory elements response to salicylic acid (SA). CGTCA-motif and TGACG-motif are the *cis*-elements response to methyl jasmonate (MeJA) [66,67,68,69,70,71,72]. These elements could be found in the most of *OsPEBP* promoters, suggesting that *OsPEBPs* may be activated by phytohormones, such as ABA, SA, and MeJA and may be involved in their signaling (Figure 7a). To explore if *OsPEBPs* are induced by ABA and MeJA, the microarray data from the Rice Expression Profile Database (RiceXPro, http://ricexpro.dna.affrc.go.jp/, accessed on 13 May 2021) and qRT-PCR were applied. The data of RiceXPro show that the expression of *OsFTL14*, *OsRCN1*, *OsRCN2*, *OsRCN3*, *OsMFT1*, *OsFTL1/OsFTL*, *OsFTL2/Hd3a*, *OsFTL13*, *OsFTL3/RFT1*, *OsMFT2*, *OsRCN4,* and *OsFTL12* is induced by ABA treatment. While the expression *OsFTL6* is downregulated by ABA treatment (Figure 7c). Consistently, the results of qRT-PCR show that the expression of *OsFTL2/Hd3a*, *OsFTL3/RFT1*, *OsFTL4*, *OsFTL8*, *OsFTL13*, *OsRCN1*, *OsRCN2*, *OsRCN3*, *OsRCN4*, and *OsMFT1* is induced, whereas the expression of *OsFTL1* and *OsFTL1* is downregulated by ABA treatment (Figure 7d). The data of RiceXPro show that the expression of *OsMFT1*, *OsFTL14*, *OsFTL12*, *OsFTL13*, and *OsFTL3/RFT1* is induced, whereas the expression of *OsFTL6* and *OsRCN1* is downregulated by JA treatment (Figure 7e). The results of qRT-PCR show that the expression of *OsFTL3/RFT1*, *OsFTL4*, *OsFTL7*, *OsFTL8*, *OsFTL10*, *OsFTL12*, *OsFTL13*, *OsFTL14*, *OsRCN2*, *OsRCN4*, and *OsMFT1* is induced, whereas the expression of *OsRCN1* and *OsMFT2* is downregulated by JA treatment (Figure 7f).

**Figure 8:**
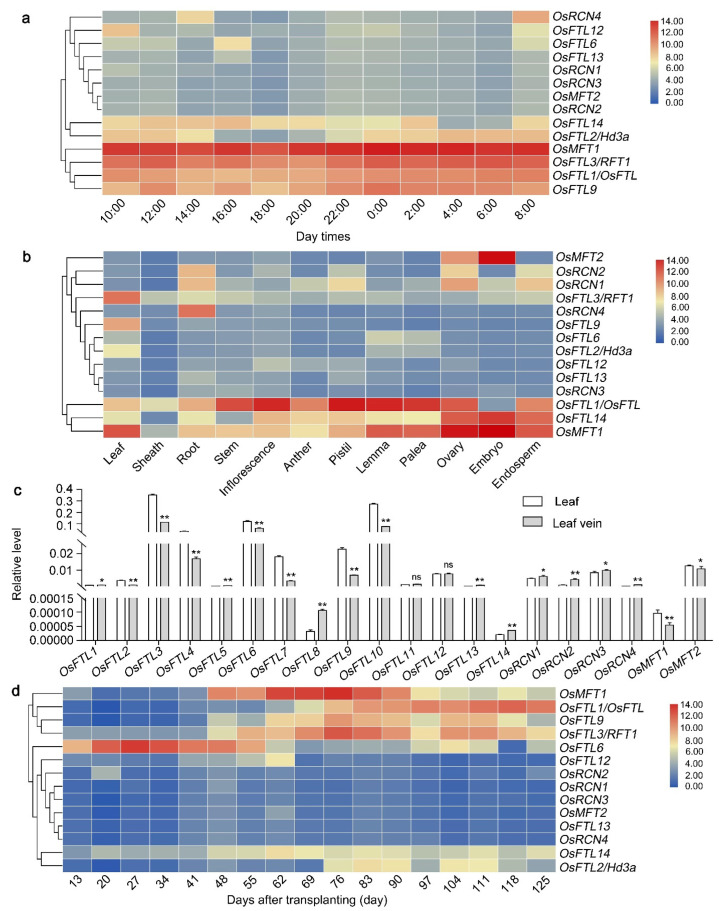
Expression profiles of *OsPEBP* genes in rice. (**a**) Expression of *OsPEBPs* under natural photoperiod. (**b**) Differential expression of *OsPEBPs* in organs, including leaf, leaf sheath, root, stem, inflorescence, anther, pistil, lemma, palea, ovary, embryo, and endosperm. (**c**) Expression of *OsPEBPs* in leaf and leaf vein of rice. (**d**) Expression of *OsPEBPs* in developmental stages. The blue to red color of the bars in **d** represents the lower to higher expression of *OsPEBPs*. Data in **c** are the mean ± SD. Statistic differences are analyzed by using a two-tailed unpaired *t* test with Welch’s correction (*, *p* < 0.05; **, *p* < 0.01).

The *cis*-acting elements response to light, including G-box and Box-4 [73], are localized in the promoter regions of *OsPEBPs*, indicating that *OsPEBPs* expression may be regulated by light (Figure 7a). To study whether OsPEBPs are involved in response to light, the diurnal enrichment of *OsPEBPs* was studied with the data from the RiceXPro database. Samples corresponding to the fully expanded flag leaves are collected in a 24-hr period at 2-hr intervals at floral transition stages during the cultivation season. The transcript level of PEBP genes, including *OsRCN4*, *OsFTL12*, *OsFTL6*, *OsFTL13*, *OsRCN1*, *OsRCN3*, *OsMFT2*, *OsRCN2*, *OsFTL14*, *OsFTL2/Hd3a*, *OsFTL3/OsRFT1*, and *OsFTL1/OsFTL* is induced by light or peaks in the early morning or morning. The expression of *OsMFT1* is constitutive (Figure 8a).

### 2.6. Temporal and Spatial Expression Pattern of PEBP Genes in Rice

The gene expression profiles are often associated with their functions. To evaluate roles of *OsPEBP* genes in regulating development, the expression profiles of *OsPEBPs* were examined with microarray data in RiceXPro database. The *OsPEBP* genes are expressed in all organs and tissues detected; but the enrichment of the transcripts are differentiated (Figure 8b). The expression of *OsMFT1*, *OsFTL14*, and *OsFTL1/OsFTL* in all organs detected is higher than other genes. The expression of *OsFTL2/Hd3a*, *OsFTL9*, *OsFTL3/RFT1*, and *OsMFT1* in leaf is highest among the organs detected (Figure 8b).

To further distinguish the differential expression of *OsPEBPs* in leaf and leaf vein, the differential expression of *OsPEBPs* was examined by qRT-PCR. The expression of the *OsPEBPs*, including *OsFTL2/Hd3a*, *OsFTL3/RFT1*, *OsFTL4*, *OsFTL6*, *OsFTL7*, *OsFTL9*, *OsFTL10*, *OsMFT1*, and *OsMFT2* in leaf vein is lower than that in leaf. The expression of the *OsPEBPs*, such as *OsFTL1/OsFTL*, *OsFTL5*, *OsFTL8*, *OsFTL11*, *OsFTL13*, *OsFTL14*, *OsRCN1*, *OsRCN2*, *OsRCN3*, and *OsRCN4* in leaf vein is higher than that in leaf (Figure 8c).

The expression patterns of *OsPEBP* genes at different developmental stages of rice were also investigated (Figure 8d). *OsPEBPs* are expressed in all the stages, which could be divided into four clusters according to their expression (Figure 4). The expression of *OsMFT1*, *OsFTL1/OsFTL*, *OsFTL9,* and *OsFTL3/RFT1* in cluster I is the highest. The expression of *OsFTL6* before 62 DAT in cluster II is higher than that after 62 day after transplanting (DAT). The expression of *OsFTL12*, *OsRCN2*, *OsRCN1*, *OsRCN3, OsMFT2, OsFTL13,* and *OsRCN4* during life cycle in cluster III is lower than other genes. The expression of *OsFTL14* and *OsFTL2/Hd3a* genes in cluster IV and genes in cluster I increases after 41 DAT (Figure 8d). The differential expression of *OsPEBPs* during different developmental stages may associated with their function.

In summary, the *OsPEBP* gene expressions are differential, revealing that *OsPEBP* genes may have different functions in regulating development and adaptation to the changes of environment of rice.

## 3. Discussion

In plants, PEBP-domain-containing proteins may act as key factors in multiple developmental processes [16,31,33,34,52]. However, most of the OsPEBPs’ functions in rice remain unclear. Exploring evolutionary features and expression profiles of OsPEBPs will be beneficial for further understanding the functions of PEBPs in rice.

Tandem and fragment duplications affect the formation of gene families [74,75]. In these studies, twenty PEBP genes are found in rice genome (Table S1). The OsPEBP genes are not evenly scattered on the rice chromosome (Figure 1). Two small gene clusters exhibit on chromosome 1 and 6. Four collinear pairs of OsPEBPs in rice are identified by syntenic analysis, suggesting OsPEBP genes undergo segmental duplications (Figure 2). These properties may imply the functional similarity and difference among OsPEBPs.

OsPEBP proteins are divided into six subgroups by phylogenetic analysis. The structure of OsPEBP genes is conserved. Most of the OsPEBP genes contain three introns and four exons. All the proteins encoded contain a PEBP domain. The key residuals in PEBPs’ C-terminal, including Glu217 (E217), Trp252 (W252), Gln254 (Q254), and Asn266 (N266), vary among OsPEBP proteins (Figure 3). These results suggest that OsPEBPs may have diverse functions and the OsPEBPs in the same subgroup may have similar functions. Consistently, OsFTL2/Hd3a and OsFTL3/RFT1 in subgroup V promote floral transition. OsRCNs in subgroup II suppress flowering. Overexpression of four OsRCNs delay floral transition [24,34,52,55,58]. OsFTL2/Hd3a regulates shoot branching of rice [58]. OsRCN knockdown plants exhibit small panicles with reduced branches [51,76,77]. However, the findings on OsPEBP genes are limited. More efforts will be put to dissect the functions of PEBP gene family in rice and other crops.

In this study, the number of PEBP colinear pairs between rice and maize, wheat, millet, brachypodium, and sorghum is higher than that between rice and soybean, barley, and Arabidopsis which are consistent with the phylogenetic relationship between rice and the other plants (Figure 6a–h). The *PEBP* genes of monocotyledon species are phylogenetically closed to rice.

Cultivated rice is domesticated from wild rice, such as *Oryza rufipogon* and *Oryza nivara* [78,79]. The genomes of wild rice species are more diverse than that of cultivated rice, suggesting that wild rice are invaluable germplasm resources for rice breeding [80]. OsPEBPs in cultivated rice have significant syntenic relationships with wild rice. About 33 PEBP pairs are identified between rice and wild rice (Figure 6i–q). The number and chromosome location of PEBP proteins in wild rice are higher than that in cultivated rice. Most of OsPEBPs are paired to more than two chromosome regions in wild rice (Figure 6i–q).

Usually, genes in a subgroup of phylogenetic tree have similar functions [81]. In this study, the phylogenetic tree of 262 PEBP-domain-containing proteins in the rice and other species was built. The PEBP orthologs from monocotyledon and dicotyledon are clustered in the same branch (Figure 4). Twenty conserved motifs are found in PEBP proteins. PEBP proteins in the same group are with similar motifs (Figure S1). These results indicate that PEBP genes are more ancient than the divergence of monocotyledon and dicotyledon plants.

The *cis*-acting regulatory elements in the promoter regions regulate gene expression and function. These *cis*-acting elements response to stress, hormone, light, and circadian clock are shared by most of *OsPEBPs* (Figure 7a). The expression of several *OsPEBPs* is induced by NaCl, ABA, JA, and light (Figure 7b–f and Figure 8a). These results suggest that OsPEBPs may be involved in flowering time control, seed dormancy, seed germination, photo-response, hormone response, and abiotic stress tolerance, especially water logging stress, in rice. The differentiation of microarray and RT-qPCR data is due to different sampling strategies. Whether *OsPEBPs* are involved in these processes of rice will be dissected.

Spatiotemporal gene expression is usually related with gene functions [82]. All *OsPEBPs* are expressed in organs detected, developmental stages, especially response to ABA and JA hormones; however, the expression of them is differential, suggesting their functional diversity (Figure 8b–d).

## 4. Materials and Methods

### 4.1. Sequence Identification of PEBP Gene Family

Full-length amino acid sequence of FT, Hd3a, and RFT1 from Arabidopsis and rice was used as queries sequences to blast against rice genome to identify PEBP genes. The Hidden Markov Model (HMM) of PEBP (PF01161) domain was downloaded from the Pfam database (http://pfam.xfam.org/, accessed on 8 April 2021). The rice protein sequence, genome, and CDS sequence were from the database of EnsemblPlants (http://plants.ensembl.org/index.html, accessed on 12 April 2021) [83,84,85,86,87,88,89].

The candidate PEBP proteins were identified by the Simple HMM search based on the TBtools [90]. The sequences of PEBP domain were obtained and used to build a rice specific HMM. The rice specific HMM was used to obtain candidate OsPEBP proteins. The candidate proteins with E-value < 0.01 were chosen. The Pfam and InterPro databases (http://www.ebi.ac.uk/interpro/, accessed on 15 April 2021) was used to verify the PEBP proteins obtained as described previously [91].

### 4.2. Characterization of OsPEBP Genes

The chromosomal locations of *OsPEBP* genes were mapped to the rice chromosome with TBtools. The PEBP gene structures of rice were analyzed by comparing the genomic sequences against the coding sequences using the TBtools [90]. The isoelectric points and molecular weights of the PEBP family proteins were calculated with ExPASy (http://expasy.org/, accessed on 18 April 2021).

### 4.3. Conserved Motifs Characterization

To identify conserved motifs in the PEBP protein family, the MEME program (http://meme-suite.org/, accessed on 19 April 2021) was applied using the following parameters: any number of repetitions; minimum 6 motifs; maximum 50 motifs; optimum 10–200 amino acids; expected E-value < 1*10-48 [92].

### 4.4. Phylogenetic Analysis

A multiple alignment of the PEBP protein amino acid sequences in rice was conducted using MEGA X (https://www.megasoftware.net/, accessed on 18 April 2021). Unrooted phylogenetic trees were built with the maximum likelihood (ML) method. Parameters, including Poisson correction, pairwise deletion, 1000 bootstrap replicates were used during the analysis. Default parameters of MCScanX were used to examine duplication of genes [93]. TBtools was used to analyze the orthologs of the PEBP genes between rice and other species.

### 4.5. Cis-Acting Element Analysis

Promoters of *OsPEBPs* were found from the Phytozome database (https://phytozome.jgi.doe.gov/, accessed on 18 April 2021). The PlantCARE database (http://bioinformatics.psb.ugent.be/webtools/plantcare/html/, accessed on 11 May 2021) was used to analyze the *cis*-acting regulatory elements in *OsPEBPs’* promoters [94].

### 4.6. Expression Analysis

Two-week-old seedlings of Shen Nong 9816 (SN9816) grown in 14 h light and 10 h dark were collected. The harvested seedlings were treated with 0 and 250 mM NaCl; 0 and 100 μM ABA; 0 and 100 μM JA for 6 h. Two biological replications were performed for each treatment. Total RNA of the samples was isolated using Takara RNAiso Plus (Takara Bio Inc., Otsu, Japan). The contimanate DNA was removed by using RQ1 RNase-Free DNase treatment (Promega, Madison, WI, USA). Then, 3 μg of treated RNA was used to synthesize the first-strand cDNA with the RevertAid First Strand cDNA Synthesis Kit (Fermentas, Waltham, MA, USA). SYBR Premix Ex Taq (Takara Bio Inc., Shiga, Japan) and a Q5 Real-Time PCR Instrument (Applied Biosystems, Waltham, MA, USA) were used for qRT-PCR [95,96]. Primers used for qRT-PCR are listed (Table S2).

The Mircroarray data are from the Rice Expression Profile Database (RiceXPro, http://ricexpro.dna.affrc.go.jp/, accessed on 13 May 2021). Four-leaf-old seedlings of rice were treated with 50 μM ABA and 100 μM MeJA for 0, 1, 3, 6, and 12 h; two biological replicates were performed. The shoot of the treated seedlings was used for microarray analysis. The signal intensity detected during hybridization was used for drawing heatmap with TBtools.

## 5. Conclusions

In summary, twenty *OsPEBPs* are found in the rice genome. The feature of the gene structure and domains and motifs of the protein encoded are analyzed. Phylogenetic trees of OsPEBP proteins and the orthologs from other crops are built, which divide the protein into six subfamily or groups. Four collinear pairs identified by synteny analysis are found between *OsPEBPs* in rice, indicating that *OsPEBPs* are subject to segmental duplication. More collinear pairs are identified among orthologs in crops. Several cis-acting elements in the promoter region of *OsPEBPs* are characterized. The expression of *OsPEBPs* is differential in organs and developmental stages and is induced by NaCl, ABA, JA, and light. The results of this study lay sound foundation to further uncover the *PEBP* gene functions and to improve varieties of crops.

## Data Availability

All data generated or analyzed during this study are included in this published article and its supplementary information files.

## Supplementary Materials

The following supporting information can be downloaded at: https://www.mdpi.com/article/10.3390/plants11121576/s1, Figure S1: Motifs identified in 262 PEBP-domain-containing proteins of crops. Table S1: Characteristics of *PEBP* genes in rice genome. Table S2: Primers used in the study.

## References

[1] Al-Mulla F., Bitar M.S., Taqi Z., Yeung K.C. (2013). RKIP: Much more than Raf kinase inhibitory protein. J. Cell. Physiol..

[2] Rajkumar K., Nichita A., Anoor P.K., Raju S., Singh S.S., Burgula S. (2016). Understanding perspectives of signalling mechanisms regulating PEBP1 function. Cell. Biochem. Funct..

[3] Wickland D.P., Hanzawa Y. (2015). The flowering locus t/terminal flower 1 Gene Family: Functional Evolution and Molecular Mechanisms. Mol. Plant.

[4] Banfield M.J., Barker J.J., Perry A.C., Brady R.L. (1998). Function from structure? The crystal structure of human phosphatidylethanolamine-binding protein suggests a role in membrane signal transduction. Structure.

[5] Serre L., Vallee B., Bureaud N., Schoentgen F., Zelwer C. (1998). Crystal structure of the phosphatidylethanolamine-binding protein from bovine brain: A novel structural class of phospholipid-binding proteins. Structure.

[6] Yeung K., Seitz T., Li S., Janosch P., McFerran B., Kaiser C., Fee F., Katsanakis K.D., Rose D.W., Mischak H. (1999). Suppression of Raf-1 kinase activity and MAP kinase signalling by RKIP. Nature.

[7] Escara-Wilke J., Yeung K., Keller E.T. (2012). Raf kinase inhibitor protein (RKIP) in cancer. Cancer Metastasis Rev..

[8] Tang H., Park S., Sun S.C., Trumbly R., Ren G., Tsung E., Yeung K.C. (2010). RKIP inhibits NF-kappaB in cancer cells by regulating upstream signaling components of the IkappaB kinase complex. FEBS Lett..

[9] Maresch J., Birner P., Zakharinov M., Toumangelova-Uzeir K., Natchev S., Guentchev M. (2011). Additive effect on survival of Raf kinase inhibitor protein and signal transducer and activator of transcription 3 in high-grade glioma. Cancer.

[10] Wall A.A., Luo L., Hung Y., Tong S.J., Condon N.D., Blumenthal A., Sweet M.J., Stow J.L. (2017). Small GTPase Rab8a-recruited Phosphatidylinositol 3-Kinase gamma Regulates Signaling and Cytokine Outputs from Endosomal Toll-like Receptors. J. Biol. Chem..

[11] Schoentgen F., Jonic S. (2020). PEBP1/RKIP behavior: A mirror of actin-membrane organization. Cell. Mol. Life Sci..

[12] Navarro C., Abelenda J.A., Cruz-Oro E., Cuellar C.A., Tamaki S., Silva J., Shimamoto K., Prat S. (2011). Control of flowering and storage organ formation in potato by FLOWERING LOCUS T. Nature.

[13] Lee R., Baldwin S., Kenel F., McCallum J., Macknight R. (2013). FLOWERING LOCUS T genes control onion bulb formation and flowering. Nat. Commun..

[14] Leeggangers H., Rosilio-Brami T., Bigas-Nadal J., Rubin N., van Dijk A.D.J., Nunez de Caceres Gonzalez F.F., Saadon-Shitrit S., Nijveen H., Hilhorst H.W.M., Immink R.G.H. (2018). Tulipa gesneriana and Lilium longiflorum PEBP Genes and Their Putative Roles in Flowering Time Control. Plant Cell. Physiol..

[15] Ben Michael T.E., Faigenboim A., Shemesh-Mayer E., Forer I., Gershberg C., Shafran H., Rabinowitch H.D., Kamenetsky-Goldstein R. (2020). Crosstalk in the darkness: Bulb vernalization activates meristem transition via circadian rhythm and photoperiodic pathway. BMC Plant Biol..

[16] Corbesier L., Vincent C., Jang S., Fornara F., Fan Q., Searle I., Giakountis A., Farrona S., Gissot L., Turnbull C. (2007). FT protein movement contributes to long-distance signaling in floral induction of Arabidopsis. Science.

[17] Abelenda J.A., Bergonzi S., Oortwijn M., Sonnewald S., Du M., Visser R.G.F., Sonnewald U., Bachem C.W.B. (2019). Source-Sink Regulation Is Mediated by Interaction of an FT Homolog with a SWEET Protein in Potato. Curr. Biol..

[18] Perilleux C., Bouche F., Randoux M., Orman-Ligeza B. (2019). Turning Meristems into Fortresses. Trends Plant Sci..

[19] Prusinkiewicz P., Erasmus Y., Lane B., Harder L.D., Coen E. (2007). Evolution and development of inflorescence architectures. Science.

[20] Lifschitz E., Ayre B.G., Eshed Y. (2014). Florigen and anti-florigen—A systemic mechanism for coordinating growth and termination in flowering plants. Front. Plant Sci..

[21] Karlgren A., Gyllenstrand N., Kallman T., Sundstrom J.F., Moore D., Lascoux M., Lagercrantz U. (2011). Evolution of the PEBP gene family in plants: Functional diversification in seed plant evolution. Plant Physiol..

[22] Abe M., Kobayashi Y., Yamamoto S., Daimon Y., Yamaguchi A., Ikeda Y., Ichinoki H., Notaguchi M., Goto K., Araki T. (2005). FD, a bZIP protein mediating signals from the floral pathway integrator FT at the shoot apex. Science.

[23] Wigge P.A., Kim M.C., Jaeger K.E., Busch W., Schmid M., Lohmann J.U., Weigel D. (2005). Integration of spatial and temporal information during floral induction in Arabidopsis. Science.

[24] Tamaki S., Matsuo S., Wong H.L., Yokoi S., Shimamoto K. (2007). Hd3a protein is a mobile flowering signal in rice. Science.

[25] Kardailsky I., Shukla V.K., Ahn J.H., Dagenais N., Christensen S.K., Nguyen J.T., Chory J., Harrison M.J., Weigel D. (1999). Activation tagging of the floral inducer FT. Science.

[26] Kobayashi Y., Kaya H., Goto K., Iwabuchi M., Araki T. (1999). A pair of related genes with antagonistic roles in mediating flowering signals. Science.

[27] Bradley D., Ratcliffe O., Vincent C., Carpenter R., Coen E. (1997). Inflorescence commitment and architecture in Arabidopsis. Science.

[28] Song Y.H., Shim J.S., Kinmonth-Schultz H.A., Imaizumi T. (2015). Photoperiodic flowering: Time measurement mechanisms in leaves. Annu. Rev. Plant Biol..

[29] Andres F., Coupland G. (2012). The genetic basis of flowering responses to seasonal cues. Nat. Rev. Genet..

[30] Conti L., Bradley D. (2007). Terminal flower1 is a mobile signal controlling Arabidopsis architecture. Plant Cell..

[31] Hanano S., Goto K. (2011). Arabidopsis TERMINAL FLOWER1 is involved in the regulation of flowering time and inflorescence development through transcriptional repression. Plant Cell..

[32] Ho W.W., Weigel D. (2014). Structural features determining flower-promoting activity of Arabidopsis flowering locus t. Plant Cell..

[33] Taoka K., Ohki I., Tsuji H., Furuita K., Hayashi K., Yanase T., Yamaguchi M., Nakashima C., Purwestri Y.A., Tamaki S. (2011). 14-3-3 proteins act as intracellular receptors for rice Hd3a florigen. Nature.

[34] Kaneko-Suzuki M., Kurihara-Ishikawa R., Okushita-Terakawa C., Kojima C., Nagano-Fujiwara M., Ohki I., Tsuji H., Shimamoto K., Taoka K.I. (2018). TFL1-Like Proteins in Rice Antagonize Rice FT-Like Protein in Inflorescence Development by Competition for Complex Formation with 14-3-3 and FD. Plant Cell. Physiol..

[35] Zhu Y., Klasfeld S., Jeong C.W., Jin R., Goto K., Yamaguchi N., Wagner D. (2020). Terminal flower 1-fd complex target genes and competition with flowering locus t. Nat. Commun..

[36] Ahn J.H., Miller D., Winter V.J., Banfield M.J., Lee J.H., Yoo S.Y., Henz S.R., Brady R.L., Weigel D. (2006). A divergent external loop confers antagonistic activity on floral regulators FT and TFL1. EMBO J..

[37] Hanzawa Y., Money T., Bradley D. (2005). A single amino acid converts a repressor to an activator of flowering. Proc. Natl. Acad. Sci. USA.

[38] Pin P.A., Benlloch R., Bonnet D., Wremerth-Weich E., Kraft T., Gielen J.J., Nilsson O. (2010). An antagonistic pair of FT homologs mediates the control of flowering time in sugar beet. Science.

[39] Abelenda J.A., Navarro C., Prat S. (2011). From the model to the crop: Genes controlling tuber formation in potato. Curr. Opin. Biotechnol..

[40] Plantenga F.D.M., Bergonzi S., Abelenda J.A., Bachem C.W.B., Visser R.G.F., Heuvelink E., Marcelis L.F.M. (2019). The tuberization signal StSP6A represses flower bud development in potato. J. Exp. Bot..

[41] Teo C.J., Takahashi K., Shimizu K., Shimamoto K., Taoka K.I. (2017). Potato Tuber Induction is Regulated by Interactions Between Components of a Tuberigen Complex. Plant Cell. Physiol..

[42] Zhang X., Campbell R., Ducreux L.J.M., Morris J., Hedley P.E., Mellado-Ortega E., Roberts A.G., Stephens J., Bryan G.J., Torrance L. (2020). TERMINAL FLOWER-1/CENTRORADIALIS inhibits tuberisation via protein interaction with the tuberigen activation complex. Plant J..

[43] Kuhn C., Grof C.P. (2010). Sucrose transporters of higher plants. Curr. Opin. Plant Biol..

[44] Jeena G.S., Kumar S., Shukla R.K. (2019). Structure, evolution and diverse physiological roles of SWEET sugar transporters in plants. Plant Mol. Biol..

[45] Morris W.L., Alamar M.C., Lopez-Cobollo R.M., Castillo Canete J., Bennett M., Van der Kaay J., Stevens J., Kumar Sharma S., McLean K., Thompson A.J. (2019). A member of the terminal flower 1/centroradialis gene family controls sprout growth in potato tubers. J. Exp. Bot..

[46] Jung C., Muller A.E. (2009). Flowering time control and applications in plant breeding. Trends Plant Sci..

[47] Srikanth A., Schmid M. (2011). Regulation of flowering time: All roads lead to Rome. Cell. Mol. Life Sci..

[48] Goff S.A., Ricke D., Lan T.H., Presting G., Wang R., Dunn M., Glazebrook J., Sessions A., Oeller P., Varma H. (2002). A draft sequence of the rice genome (*Oryza sativa* L. ssp. japonica). Science.

[49] Yu J., Hu S., Wang J., Wong G.K., Li S., Liu B., Deng Y., Dai L., Zhou Y., Zhang X. (2002). A draft sequence of the rice genome (*Oryza sativa* L. ssp. indica). Science.

[50] Fang M., Zhou Z., Zhou X., Yang H., Li M., Li H. (2019). Overexpression of OsFTL10 induces early flowering and improves drought tolerance in *Oryza sativa* L. PeerJ.

[51] Nakagawa M., Shimamoto K., Kyozuka J. (2002). Overexpression of RCN1 and RCN2, rice TERMINAL FLOWER 1/CENTRORADIALIS homologs, confers delay of phase transition and altered panicle morphology in rice. Plant J..

[52] Komiya R., Ikegami A., Tamaki S., Yokoi S., Shimamoto K. (2008). Hd3a and RFT1 are essential for flowering in rice. Development.

[53] Chen Y., Shen J., Zhang L., Qi H., Yang L., Wang H., Wang J., Wang Y., Du H., Tao Z. (2021). Nuclear translocation of OsMFT1 that is impeded by OsFTIP1 promotes drought tolerance in rice. Mol. Plant.

[54] Turck F., Fornara F., Coupland G. (2008). Regulation and identity of florigen: FLOWERING LOCUS T moves center stage. Annu. Rev. Plant Biol..

[55] Komiya R., Yokoi S., Shimamoto K. (2009). A gene network for long-day flowering activates RFT1 encoding a mobile flowering signal in rice. Development.

[56] Tsuji H., Taoka K., Shimamoto K. (2011). Regulation of flowering in rice: Two florigen genes, a complex gene network, and natural variation. Curr. Opin. Plant Biol..

[57] Kobayashi K., Yasuno N., Sato Y., Yoda M., Yamazaki R., Kimizu M., Yoshida H., Nagamura Y., Kyozuka J. (2012). Inflorescence meristem identity in rice is specified by overlapping functions of three AP1/FUL-like MADS box genes and PAP2, a SEPALLATA MADS box gene. Plant Cell..

[58] Tsuji H., Tachibana C., Tamaki S., Taoka K., Kyozuka J., Shimamoto K. (2015). Hd3a promotes lateral branching in rice. Plant J..

[59] Park S.J., Jiang K., Tal L., Yichie Y., Gar O., Zamir D., Eshed Y., Lippman Z.B. (2014). Optimization of crop productivity in tomato using induced mutations in the florigen pathway. Nat. Genet..

[60] Li C., Lin H., Dubcovsky J. (2015). Factorial combinations of protein interactions generate a multiplicity of florigen activation complexes in wheat and barley. Plant J..

[61] Danilevskaya O.N., Meng X., Hou Z., Ananiev E.V., Simmons C.R. (2008). A genomic and expression compendium of the expanded PEBP gene family from maize. Plant Physiol..

[62] Hou Z., Cao J. (2016). Comparative study of the P2X gene family in animals and plants. Purinergic Signal..

[63] Cao J., Lv Y. (2016). Evolutionary analysis of the jacalin-related lectin family genes in 11 fishes. Fish. Shellfish Immunol..

[64] Park E.H., Lee H.Y., Ryu Y.W., Seo J.H., Kim M.D. (2011). Role of osmotic and salt stress in the expression of erythrose reductase in Candida magnoliae. J. Microbiol. Biotechnol..

[65] Kyozuka J., Olive M., Peacock W.J., Dennis E.S., Shimamoto K. (1994). Promoter elements required for developmental expression of the maize Adh1 gene in transgenic rice. Plant Cell..

[66] Hattori T., Totsuka M., Hobo T., Kagaya Y., Yamamoto-Toyoda A. (2002). Experimentally determined sequence requirement of ACGT-containing abscisic acid response element. Plant Cell. Physiol..

[67] Busk P.K., Pages M. (1998). Regulation of abscisic acid-induced transcription. Plant Mol. Biol..

[68] Gomez-Porras J.L., Riano-Pachon D.M., Dreyer I., Mayer J.E., Mueller-Roeber B. (2007). Genome-wide analysis of ABA-responsive elements ABRE and CE3 reveals divergent patterns in Arabidopsis and rice. BMC Genom..

[69] He Y., Gan S. (2001). Identical promoter elements are involved in regulation of the OPR1 gene by senescence and jasmonic acid in Arabidopsis. Plant Mol. Biol..

[70] Turner J.G., Ellis C., Devoto A. (2002). The jasmonate signal pathway. Plant Cell.

[71] Staswick P.E. (2008). JAZing up jasmonate signaling. Trends Plant Sci..

[72] Yang W., Zhang W., Wang X. (2017). Post-translational control of ABA signalling: The roles of protein phosphorylation and ubiquitination. Plant Biotechnol J..

[73] Giuliano G., Pichersky E., Malik V.S., Timko M.P., Scolnik P.A., Cashmore A.R. (1988). An evolutionarily conserved protein binding sequence upstream of a plant light-regulated gene. Proc. Natl. Acad. Sci. USA.

[74] Cannon S.B., Mitra A., Baumgarten A., Young N.D., May G. (2004). The roles of segmental and tandem gene duplication in the evolution of large gene families in Arabidopsis thaliana. BMC Plant Biol..

[75] Holub E.B. (2001). The arms race is ancient history in Arabidopsis, the wildflower. Nat. Rev. Genet..

[76] Liu C., Teo Z.W., Bi Y., Song S., Xi W., Yang X., Yin Z., Yu H. (2013). A conserved genetic pathway determines inflorescence architecture in Arabidopsis and rice. Dev. Cell..

[77] Wang L., Sun S., Jin J., Fu D., Yang X., Weng X., Xu C., Li X., Xiao J., Zhang Q. (2015). Coordinated regulation of vegetative and reproductive branching in rice. Proc. Natl. Acad. Sci. USA.

[78] Kovach M.J., Sweeney M.T., McCouch S.R. (2007). New insights into the history of rice domestication. Trends Genet..

[79] Sang T., Ge S. (2007). Genetics and phylogenetics of rice domestication. Curr. Opin. Genet. Dev..

[80] Tanksley S.D., McCouch S.R. (1997). Seed banks and molecular maps: Unlocking genetic potential from the wild. Science.

[81] Liu M., Ma Z., Sun W., Huang L., Wu Q., Tang Z., Bu T., Li C., Chen H. (2019). Genome-wide analysis of the NAC transcription factor family in Tartary buckwheat (*Fagopyrum tataricum*). BMC Genom..

[82] Vasav A.P., Barvkar V.T. (2019). Phylogenomic analysis of cytochrome P450 multigene family and their differential expression analysis in *Solanum lycopersicum* L. suggested tissue specific promoters. BMC Genom..

[83] Schnable P.S., Ware D., Fulton R.S., Stein J.C., Wei F., Pasternak S., Liang C., Zhang J., Fulton L., Graves T.A. (2009). The B73 maize genome: Complexity, diversity, and dynamics. Science.

[84] Mascher M., Gundlach H., Himmelbach A., Beier S., Twardziok S.O., Wicker T., Radchuk V., Dockter C., Hedley P.E., Russell J. (2017). A chromosome conformation capture ordered sequence of the barley genome. Nature.

[85] Paterson A.H., Bowers J.E., Bruggmann R., Dubchak I., Grimwood J., Gundlach H., Haberer G., Hellsten U., Mitros T., Poliakov A. (2009). The Sorghum bicolor genome and the diversification of grasses. Nature.

[86] International Brachypodium I. (2010). Genome sequencing and analysis of the model grass *Brachypodium distachyon*. Nature.

[87] Waters D.L., Nock C.J., Ishikawa R., Rice N., Henry R.J. (2012). Chloroplast genome sequence confirms distinctness of Australian and Asian wild rice. Ecol. Evol..

[88] Schmutz J., Cannon S.B., Schlueter J., Ma J., Mitros T., Nelson W., Hyten D.L., Song Q., Thelen J.J., Cheng J. (2010). Genome sequence of the palaeopolyploid soybean. Nature.

[89] Ouyang S., Zhu W., Hamilton J., Lin H., Campbell M., Childs K., Thibaud-Nissen F., Malek R.L., Lee Y., Zheng L. (2007). The TIGR Rice Genome Annotation Resource: Improvements and new features. Nucleic Acids Res..

[90] Chen C., Xia R., Chen H., He Y. (2018). TBtools, a Toolkit for Biologists integrating various biological data handling tools with a user-friendly interface. BioRxiv.

[91] Zhu M., Yan B., Hu Y., Cui Z., Wang X. (2020). Genome-wide identification and phylogenetic analysis of rice FTIP gene family. Genomics.

[92] Bailey T.L., Boden M., Buske F.A., Frith M., Grant C.E., Clementi L., Ren J., Li W.W., Noble W.S. (2009). MEME SUITE: Tools for motif discovery and searching. Nucleic Acids Res..

[93] Wang Y., Tang H., Debarry J.D., Tan X., Li J., Wang X., Lee T.H., Jin H., Marler B., Guo H. (2012). MCScanX: A toolkit for detection and evolutionary analysis of gene synteny and collinearity. Nucleic Acids Res..

[94] Lescot M., Dehais P., Thijs G., Marchal K., Moreau Y., Van de Peer Y., Rouze P., Rombauts S. (2002). PlantCARE, a database of plant cis-acting regulatory elements and a portal to tools for in silico analysis of promoter sequences. Nucleic Acids Res..

[95] Wang X., Wu F., Xie Q., Wang H., Wang Y., Yue Y., Gahura O., Ma S., Liu L., Cao Y. (2012). SKIP is a component of the spliceosome linking alternative splicing and the circadian clock in Arabidopsis. Plant Cell..

[96] Cui Z., Tong A., Huo Y., Yan Z., Yang W., Yang X., Wang X.X. (2017). SKIP controls flowering time via the alternative splicing of SEF pre-mRNA in Arabidopsis. BMC Biol..

