# Supplementary material for: Genomic Survey of PEBP Gene Family in Rice: Identification, Phylogenetic Analysis, and Expression Profiles in Organs and under Abiotic Stresses
Source: Plants (Basel). 2022 Jun 15;11(12):1576. doi: 10.3390/plants11121576 (PMC9228618; doi:10.3390/plants11121576)
Supplement: Supplementary file 1 [file plants-11-01576-s001.zip › plants-1748662-supplementary materials.pdf]

Supplementary Information

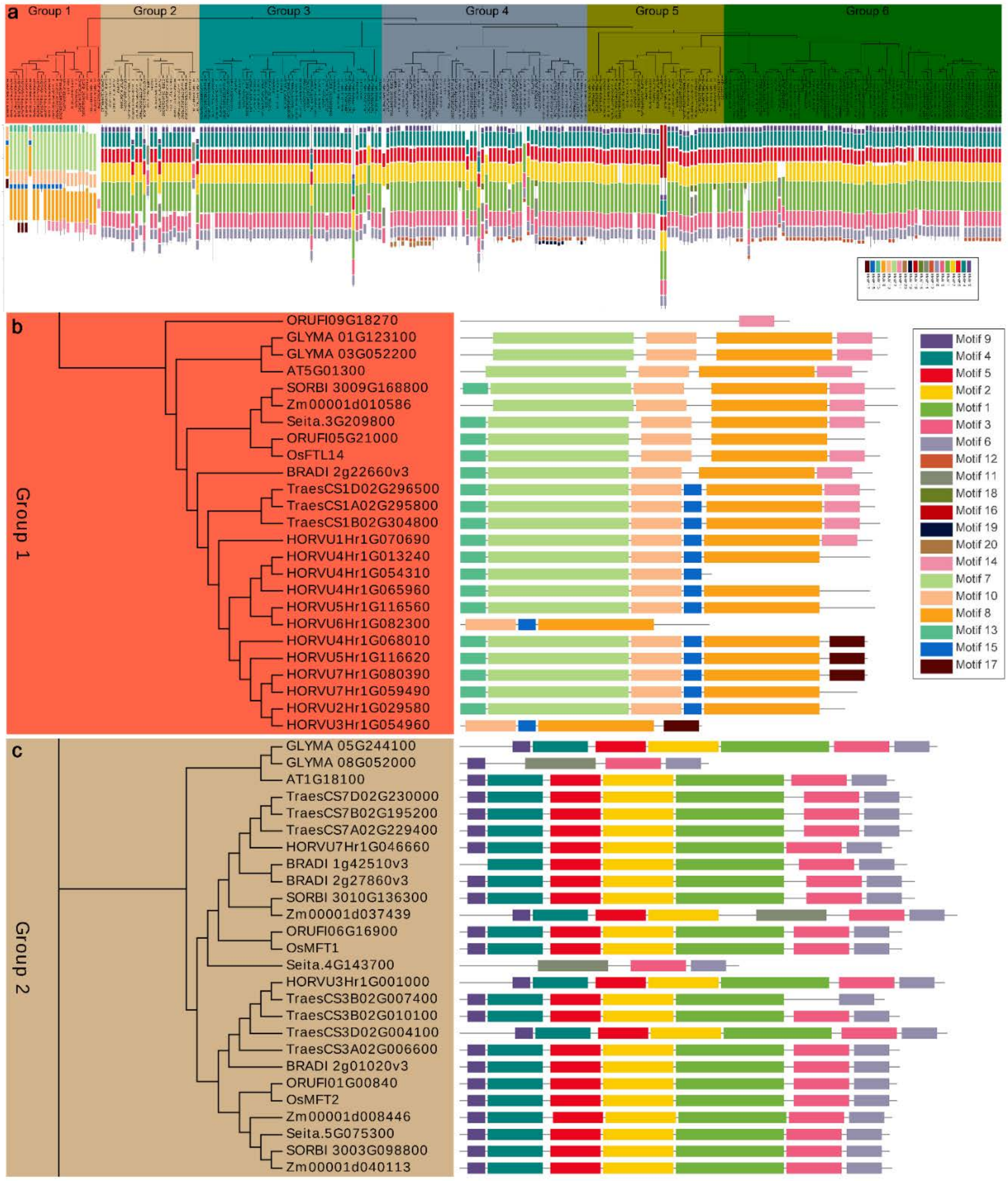

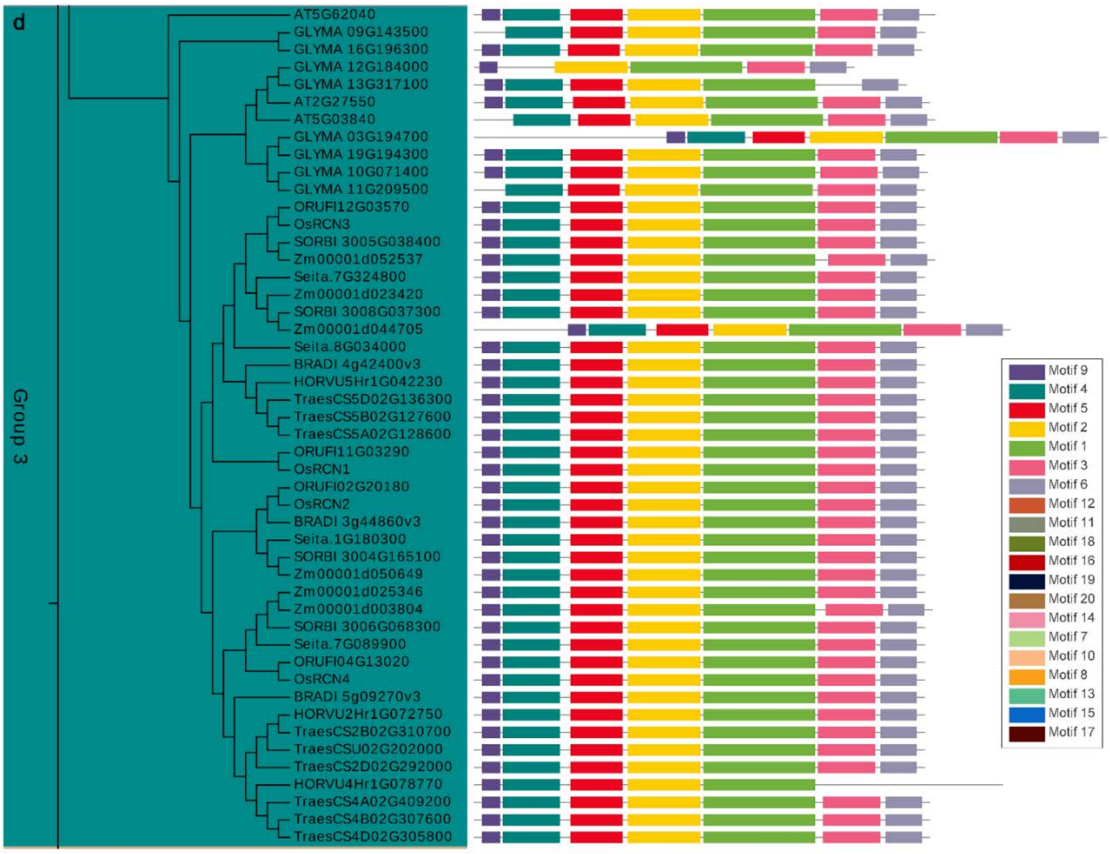
d
Group 3
AT5G62040
GLYMA 09G143500
GLYMA 16G196300
GLYMA 12G184000
GLYMA 13G317100
AT2G27550
AT5G03840
GLYMA 03G194700
GLYMA 19G194300
GLYMA 10G071400
GLYMA 11G209500
ORUFI12G03570
OsRCN3
SORBI 3005G038400
Zm00001d052537
Seita.7G324800
Zm00001d023420
SORBI 3008G037300
Zm00001d044705
Seita.8G034000
BRADI 4g42400v3
HORVU5Hr1G042230
TraesCS5D02G136300
TraesCS5B02G127600
TraesCS5A02G128600
ORUFI11G03290
OsRCN1
ORUFI02G20180
OsRCN2
BRADI 3g44860v3
Seita.1G180300
SORBI 3004G165100
Zm00001d050649
Zm00001d025346
Zm00001d003804
SORBI 3006G068300
Seita.7G089900
ORUFI04G13020
OsRCN4
BRADI 5g09270v3
HORVU2Hr1G072750
TraesCS2B02G310700
TraesCSU02G202000
TraesCS2D02G292000
HORVU4Hr1G078770
TraesCS4A02G409200
TraesCS4B02G307600
TraesCS4D02G305800
Motif 9
Motif 4
Motif 5
Motif 2
Motif 1
Motif 3
Motif 6
Motif 12
Motif 11
Motif 18
Motif 16
Motif 19
Motif 20
Motif 14
Motif 7
Motif 10
Motif 8
Motif 13
Motif 15
Motif 17

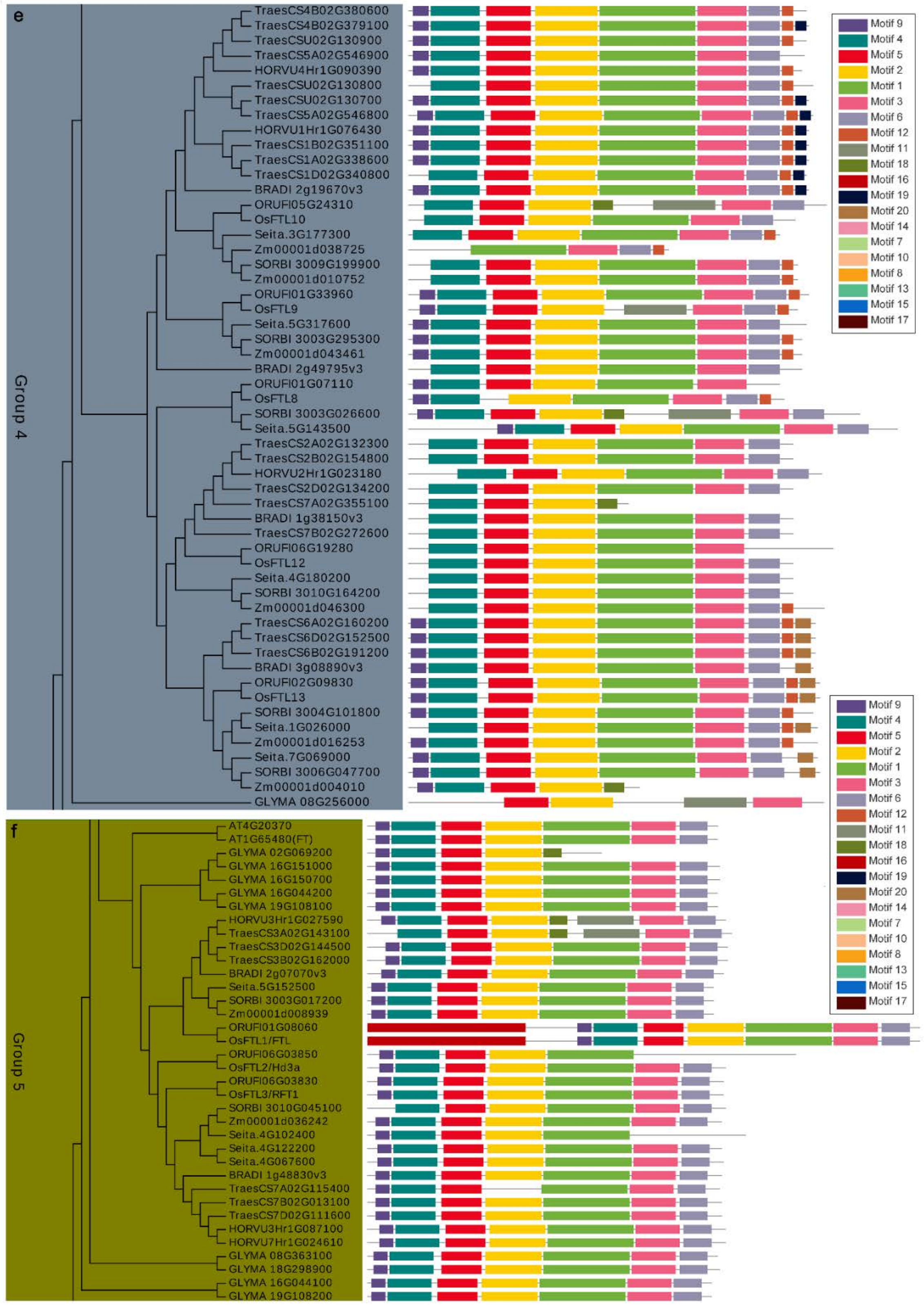
e
Group 4
TraesCS4B02G380600
TraesCS4B02G379100
TraesCSU02G130900
TraesCS5A02G546900
HORVU4Hr1G090390
TraesCSU02G130800
TraesCSU02G130700
TraesCS5A02G546800
HORVU1Hr1G076430
TraesCS1B02G351100
TraesCS1A02G338600
TraesCS1D02G340800
BRADI 2g19670v3
ORUFI05G24310
OsFTL10
Seita.3G177300
Zm00001d038725
SORBI 3009G199900
Zm00001d010752
ORUFI01G33960
OsFTL9
Seita.5G317600
SORBI 3003G295300
Zm00001d043461
BRADI 2g49795v3
ORUFI01G07110
OsFTL8
SORBI 3003G026600
Seita.5G143500
TraesCS2A02G132300
TraesCS2B02G154800
HORVU2Hr1G023180
TraesCS2D02G134200
TraesCS7A02G355100
BRADI 1g38150v3
TraesCS7B02G272600
ORUFI06G19280
OsFTL12
Seita.4G180200
SORBI 3010G164200
Zm00001d046300
TraesCS6A02G160200
TraesCS6D02G152500
TraesCS6B02G191200
BRADI 3g08890v3
ORUFI02G09830
OsFTL13
SORBI 3004G101800
Seita.1G026000
Zm00001d016253
Seita.7G069000
SORBI 3006G047700
Zm00001d004010
GLYMA 08G256000
Motif 9
Motif 4
Motif 5
Motif 2
Motif 1
Motif 3
Motif 6
Motif 12
Motif 11
Motif 18
Motif 16
Motif 19
Motif 20
Motif 14
Motif 7
Motif 10
Motif 8
Motif 13
Motif 15
Motif 17
f
Group 5
AT4G20370
AT1G65480(FT)
GLYMA 02G069200
GLYMA 16G151000
GLYMA 16G150700
GLYMA 16G044200
GLYMA 19G108100
HORVU3Hr1G027590
TraesCS3A02G143100
TraesCS3D02G144500
TraesCS3B02G162000
BRADI 2g07070v3
Seita.5G152500
SORBI 3003G017200
Zm00001d008939
ORUFI01G08060
OsFTL1/FTL
ORUFI06G03850
OsFTL2/Hd3a
ORUFI06G03830
OsFTL3/RFT1
SORBI 3010G045100
Zm00001d036242
Seita.4G102400
Seita.4G122200
Seita.4G067600
BRADI 1g48830v3
TraesCS7A02G115400
TraesCS7B02G013100
TraesCS7D02G111600
HORVU3Hr1G087100
HORVU7Hr1G024610
GLYMA 08G363100
GLYMA 18G298900
GLYMA 16G044100
GLYMA 19G108200
Motif 9
Motif 4
Motif 5
Motif 2
Motif 1
Motif 3
Motif 6
Motif 12
Motif 11
Motif 18
Motif 16
Motif 19
Motif 20
Motif 14
Motif 7
Motif 10
Motif 8
Motif 13
Motif 15
Motif 17

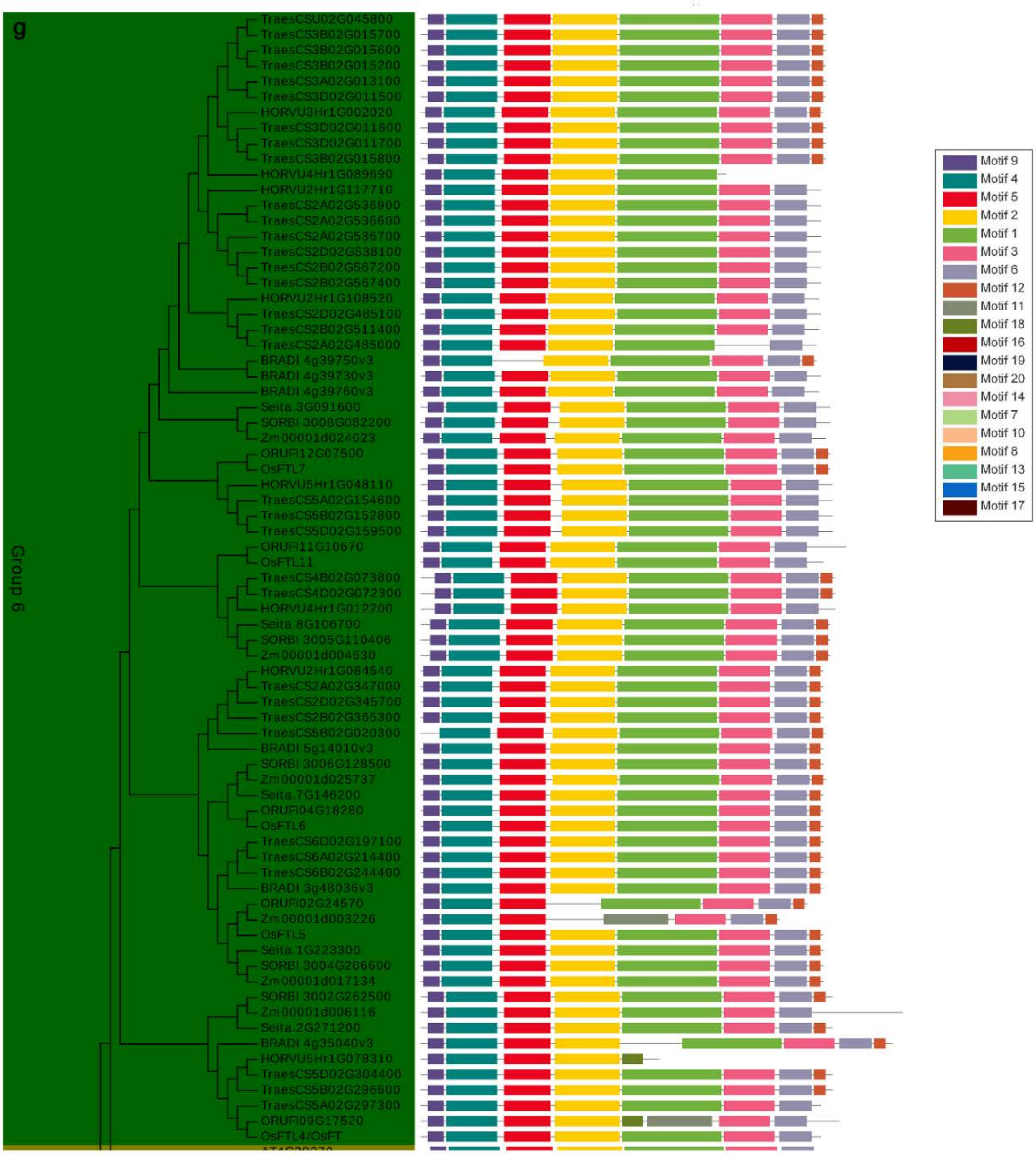

Figure S1. (a) Motifs identified in 262 PEBP-domain containing proteins of crops. (**b**) Enlarged figure of motifs identified in Group 1. (**c**) Enlarged figure of motifs identified in Group 2. (**d**) Enlarged figure of motifs identified in Group 3. (**e**) Enlarged figure of motifs identified in Group 4. (**f**) Enlarged figure of motifs identified in Group 5. (**g**) Enlarged figure of motifs identified in Group 6. Motifs are distinguished by different colors indicated in the frame.

Table S1 Characteristics of *PEBP* genes in rice genome

Gene name	MSU-ID	RAP-ID	Chromosome localization	CDS length（bp）	Exon number	Protein length	Protein MW	pI
OsFTL1/FTL	LOC_Os01g11940	Os01g0218500	Chr. 1	831	4	277	298.53	8.89
OsFTL2/Hd3a	LOC_Os06g06320	Os06g0157700	Chr. 6	540	4	180	198.39	7.73
OsFTL3/RFT1	LOC_Os06g06300	Os06g0157500	Chr. 6	537	4	179	197.07	8.52
OsFTL4/osFT	LOC_Os09g33850	Os09g0513500	Chr. 9	522	4	174	194.14	6.84
OsFTL5	LOC_Os02g39064	Os02g0602601	Chr. 2	525	4	175	196.41	8.62
OsFTL6	LOC_Os04g41130	Os04g0488400	Chr. 4	525	4	175	196.48	8.55
OsFTL7	LOC_Os12g13030	Os12g0232300	Chr. 12	534	4	178	193.74	8.51
OsFTL8	LOC_Os01g10590	Os01g0202700	Chr. 1	510	4	170	183.06	7.88
OsFTL9	LOC_Os01g54490	Os01g0748800	Chr. 1	528	4	176	196.38	7.05
OsFTL10	LOC_Os05g44180	Os05g0518000	Chr. 5	525	4	175	196.55	7.83
OsFTL11	LOC_Os11g18870	Os11g0293800	Chr. 11	525	4	175	198.93	8.5
OsFTL12	LOC_Os06g35940	Os06g0552900	Chr. 6	522	4	174	194.56	9.24
OsFTL13	LOC_Os02g13830	Os02g0232300	Chr. 2	558	4	186	203.09	6.83
OsFTL14	LOC_Os05g39250	Os05g0468800	Chr. 5	504	2	168	182.46	5.71
OsRCN1	LOC_Os11g05470	Os11g0152500	Chr. 11	522	4	174	197.37	9.16
OsRCN2	LOC_Os02g32950	Os02g0531600	Chr. 2	522	4	174	194.39	9.44
OsRCN3	LOC_Os12g05590	Os12g0152000	Chr. 12	522	4	174	195.99	8.79
OsRCN4	LOC_Os04g33570	Os04g0411400	Chr. 4	522	4	174	194.06	9.51
OsMFT1	LOC_Os06g30370	Os06g0498800	Chr. 6	531	4	177	192.07	7.94
OsMFT2	LOC_Os01g02120	Os01g0111600	Chr. 1	525	4	175	193.54	6.91

Table S2 Primers used in the study

Name	Primer sequence
OsFTL1ReF	AGTACCTGCACTGGCTGGTC
OsFTL1ReR	AGACTCCCTCTGGCAGTTGA
OsFTL2ReF	TTGGTAGGGTTGTGGGTGAT
OsFTL2ReR	GTTAGGGTCACTTGGGCTTG
OsFTL3ReF	TCGTCCGGATCACTAACCTC
OsFTL3ReR	ACCAGGAATATCGGTGACCA
OsFTL4ReF	TTGGTGACAGACATCCCAGA
OsFTL4ReR	GCAACAGGTGGTCCAAGATT
OsFTL5ReF	TGACGGACATTCCAGAAACA
OsFTL5ReR	CCGGTGGTCCTAGGTTGTAA
OsFTL6ReF	ACATTCCAGAATCGGCAAAT
OsFTL6ReR	TGGCAATTGAAGAACACTGC
OsFTL7ReF	CAACCCTTCCAAAAGGGAAT
OsFTL7ReR	GTCCCTGGTGTTGAAGTTGG
OsFTL8ReF	GACGTGGTGGACCTGTTCTC
OsFTL8ReR	GTTCGCATCAGTTGTTCCAG
OsFTL9ReF	CCAAGCAACCCATCACTAGG
OsFTL9ReR	TATGTAGCGGCCACAATGTC
OsFTL10ReF	CATCGCTGAGGGAGTACTTG
OsFTL10ReR	GTGTTGGCGTGCAAAAATTC
OsFTL11ReF	GTAATGGTGGACCCTGATGC
OsFTL11ReR	TAGGTGGTCTGCCTGACTGA
OsFTL12ReF	GGTGGATCCTGATGCTCCTA
OsFTL12ReR	TTGAAGTTCTGACGCACCTG
OsFTL13ReF	GTCCTGGATCCCTTCATCAG
OsFTL13ReR	TTCCCTCAGAGTTGGATTGC
OsFTL14ReF	GAGTACGGCGGCATCCAG
OsFTL14ReR	GCCATGAGCTCGGCTTCT
OsRCN1ReF	ATGTGCCAGGACCAAGTGAT
OsRCN1ReR	GAAATGGTCCCTGAAGGATG
OsRCN2ReF	CAACAAGCAGGTGTTCAACG
OsRCN2ReR	CTCCCAAAAGAGGCATCAGT
OsRCN3ReF	ATGTGCCAGGACCAAGTGAT
OsRCN3ReR	TCCCTAGAGGATGGCACAAC
OsRCN4ReF	AACGGCCACGAGTTATTCC
OsRCN4ReR	GCTCTCGTAGCTCACCACCT
OsMFT1ReF	GTCAACGAGCTCTTCGCTCT
OsMFT1ReR	TCTGCTGGAACAGCACCAT
OsMFT2ReF	CGACAGGGACATCAGCAAC
OsMFT2ReR	CCCCATGTACTCCACCATCT
